# Supplementary figures and images for: Matrix Metalloproteinase 2 and 9 Enzymatic Activities are Selectively Increased in the Myocardium of Chronic Chagas Disease Cardiomyopathy Patients: Role of TIMPs
Source: Front Cell Infect Microbiol. 2022 Mar 17;12:836242. doi: 10.3389/fcimb.2022.836242 (PMC8968914; doi:10.3389/fcimb.2022.836242)

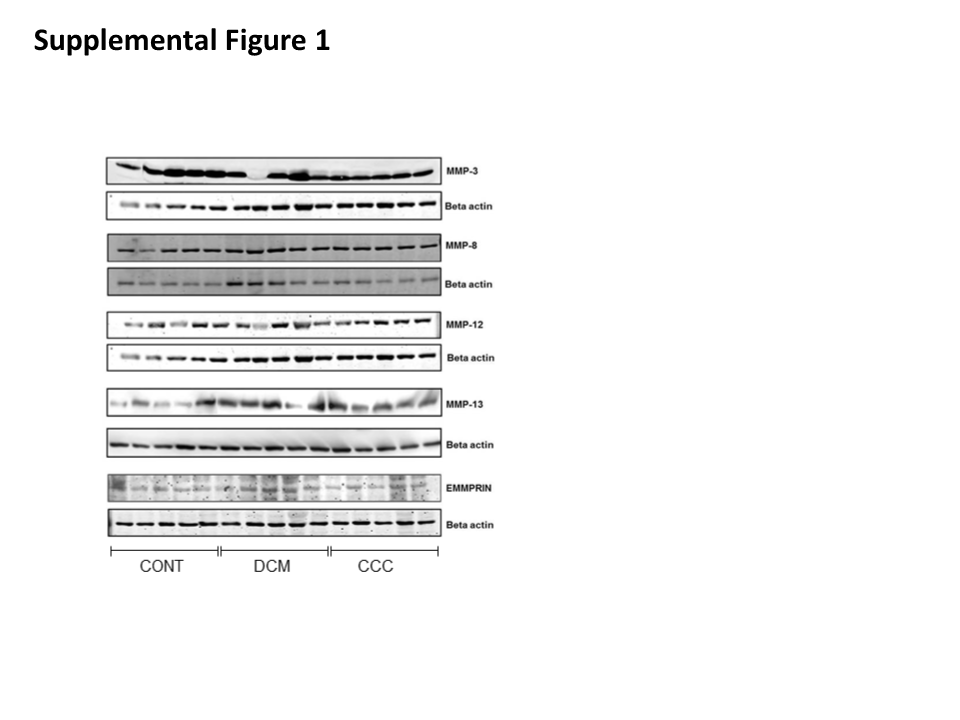

Supplement: Supplementary Figure 1 — Western blots showing MMP-3 (54 kD), -8 (65 kD), -12 (54 kD), -13 and EMMPRIN/CD147 (60 kD) protein bands. Protein bands stained with specific antibodies and developed as in Methods are depicted, together with the densitometric measurements using beta actin as a loading control. [file Image_1.tif]

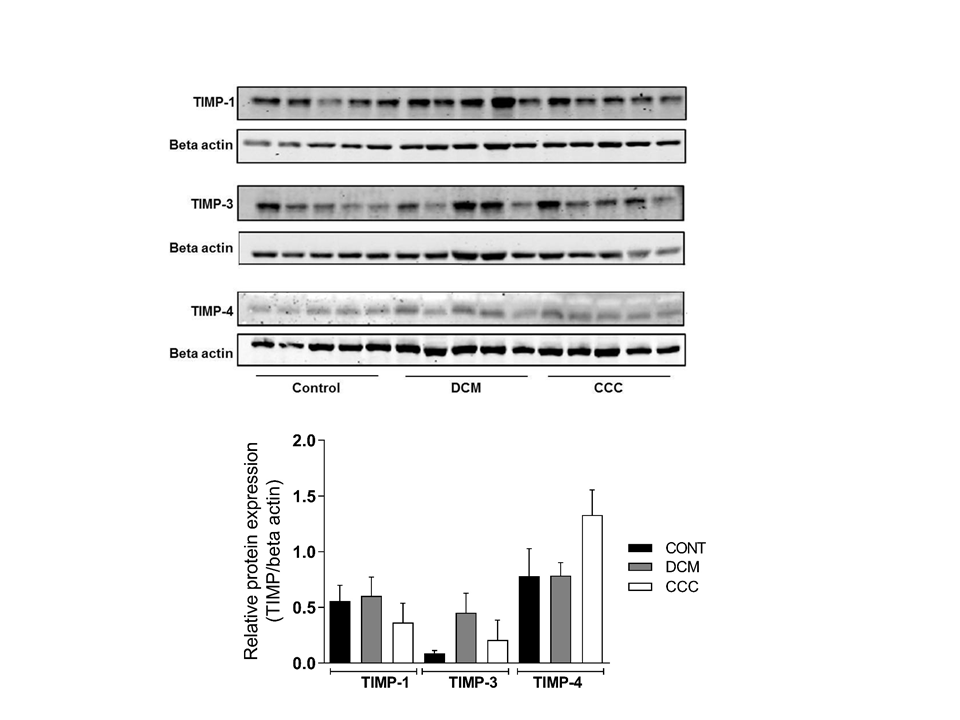

Supplement: Supplementary Figure 2 — Western blots showing TIMP-1, TIMP-3 and TIMP-4 protein bands. The densitometric values of TIMP-2 protein for each sample were normalized by the values of 42 kD Beta actin band, as described in methods section. Groups were compared by a non-parametrical test (Mann-Whitney Rank Sum Test) with GraphPad Prism software (version 6.0; GraphPad). Results were expressed as mean ± SD. [file Image_2.tif]
